# Perceptions of complementary, alternative, and integrative medicine: Insights from a large-scale international cross-sectional survey of surgery researchers and clinicians

**DOI:** 10.1016/j.heliyon.2025.e42418

**Published:** 2025-01-31

**Authors:** Jeremy Y. Ng, Brenda X. Lin, Holger Cramer

**Affiliations:** a Institute of General Practice and Interprofessional Care, University Hospital Tübingen, Tübingen, Germany; b Robert Bosch Center for Integrative Medicine and Health, Bosch Health Campus, Stuttgart, Germany

**Keywords:** Complementary and alternative medicine, Integrative medicine, Patient care, Perceptions, Surgery, Survey

## Abstract

**Background:**

Given the potential benefits of complementary, alternative, and integrative medicine (CAIM) in perioperative care and the high prevalence of their use alongside conventional treatments, understanding perceptions of CAIM during surgery is important.

**Methods:**

A survey was conducted among authors who published in surgery journals. 40 074 clinicians and researchers were invited to participate. The survey included questions about demographics and CAIM perceptions.

**Results:**

The survey received 599 responses, with most identifying as both researchers and clinicians (n = 331, 55.3 %). Mind-body therapies (n = 212, 47.1 %) were considered the most promising CAIM areas for surgery. Most respondents believed CAIM therapies are safe (n = 184, 46.2 %) but were uncertain about their effectiveness (n = 153, 38.6 % disagreed and n = 169, 42.7 % were neutral). Many agreed on the value of CAIM research (n = 310, 77.9 %), increased funding (n = 224, 55.9 %), and clinician training through formal (n = 215, 52.9 %) and supplementary (n = 246, 61.8 %) education.

**Conclusions:**

Surgery clinicians and researchers show strong interest in more CAIM education and research. This study's findings can guide the development of resources and training programs to improve CAIM knowledge and perceptions.

## Highlights

• Mind-body therapies were seen as the most promising CAIM area.
• Most respondents agreed that there is value in conducting CAIM research.
• Researchers and clinicians have higher confidence in CAIM safety than efficacy.
• More CAIM training through formal or supplemental education is necessary.

## 1 Background

Surgery is a branch of medicine that is focused on treating injuries, illnesses, and disorders using manual and instrumental methods [1]. There are many surgical specialities, which include but are not limited to general surgery, plastic surgery, orthopaedic surgery, and thoracic surgery [1]. A large research focus in the field of surgery surrounds care during the perioperative period, which is defined as the time period between the contemplation of surgery and recovery [2]. The perioperative period encompasses the preoperative phase, the intraoperative phase, and the postoperative phase [3]. Many patients who undergo surgery experience anxiety in the preoperative and postoperative periods [4], which is directly correlated to a number of factors, including administration of anaesthetic agents, anxiety surrounding the surgery itself, or perceived postoperative pain [5]. Moreover, postoperative pain, stress, and anxiety frequently remains severe despite the drug treatment [5]. While often effective, there are also limitations to pharmaceutical medications, such as concerns about addiction to pain medication [6], negative side effects, and incomplete efficacy [7]. Research has also found that patients are interested in taking a more active role in their postoperative recovery and believe that personal attitudes (e.g., maintaining a positive mindset) and self-directed healing (e.g., through mind-body therapies or natural product usage) has a positive impact on recovery [8]. As a result, some patients are turning to complementary, alternative, and integrative medicine (CAIM) therapies to integrate more self-management in the healing process and supplement their conventional postoperative pain and recovery treatments [8]. Among patients across a range of surgical disciplines, the use of CAIM is widespread with studies indicating a prevalence of 23.0 %–65.9 % to supplement their conventional perioperative care [9]. “Complementary medicine” can be defined as treatments utilized alongside conventional medicine, while “alternative medicine” replaces conventional medical approaches [10]. Examples of therapies that are used alternatively include Ayurveda and naturopathy [11]. In recent times, the field of integrative medicine has gained popularity by combining both conventional and complementary methods to offer a more comprehensive approach to healthcare [10,12,13]. In the context of this research, these approaches will all be labelled as CAIM. Studies have found that the use of aromatherapy, massage, and acupuncture helps patients relieve preoperative anxiety [14]. Additionally, it was found that the incorporation of natural products (e.g., vitamins and herbs) and mind-body therapies (e.g., meditation) alongside their prescribed treatments were beneficial to patient perceptions of postoperative recovery [8]. Due to the potential benefits of CAIM use in perioperative care and the high prevalence of patients who already use them to supplement their conventional treatments, it is important to contextualize how CAIM use during the perioperative period of surgery is perceived.

Different CAIM therapies are used by patients undergoing various subtypes of surgery. For example, patients who undergo orthopaedic and trauma surgery frequently use exercise therapies, mindfulness, and traditional Chinese medicine for pain management [15]. Common CAIM treatments that are generally used among patients who undergo surgery include herbal products, such as ginger chewing, and techniques, such as acupressure and aromatherapy for postoperative treatment of nausea and vomiting [16]. The widespread use of CAIM treatments has an impact on the periods surrounding surgery [17]. These treatments can carry potential risks, especially concerning significant side effects and adverse drug interactions, such as reduced blood clotting, when herbal medications are combined with conventional therapies in surgical care [18]. Furthermore, patients often withhold information about their CAIM use from conventional healthcare providers due to past negative experiences or fear of their providers’ preconceptions regarding CAIM [19]. Despite the potential adverse side effects of some CAIM therapies, several randomized control trials for aromatherapy [20] and massage therapy [21] have reported positive outcomes of CAIM interventions in perioperative care. Though some healthcare professionals see CAIM as a beneficial complement to traditional healthcare, others harbour doubts regarding its efficacy and safety [22]. Given the potentially serious complications associated with combining CAIM treatments with conventional medications, it is important to improve our comprehension of how surgical researchers and clinicians perceive CAIM. The perception of CAIM among surgery researchers remains largely unexplored, and there is a paucity of scholarly literature addressing this topic outside of researchers who possess a particular focus on CAIM [23]. Various studies have been conducted on the knowledge and perception of CAIM among surgery researchers and surgical care providers in Sweden [24,25] and Hungary [19]. To the best of our knowledge, no international studies collected the perceptions of surgical researchers and clinicians on CAIM.

This study seeks to investigate the perspectives of both surgical researchers and clinicians regarding CAIM through an international, cross-sectional survey study design. The findings from this survey have the potential to provide insight into the challenges and opportunities associated with the use of CAIM in perioperative care. Ultimately, this research may help to better understand surgery researchers' and clinicians’ perspectives on CAIM, which can assist in the development of educational resources for CAIM use in surgery research and practice.

## 2 Methods

### 2.1 Transparency statement

Ethics approval was granted to conduct this study from the Research Ethics Board at the University Hospital Tübingen (REB Number: 389/2023BO2). The study protocol was registered and made accessible on the Open Science Framework (OSF) and can be found here https://doi.org/10.17605/OSF.IO/538AH. The study materials and raw data have also been provided on OSF and can be found here: https://doi.org/10.17605/OSF.IO/8NUGQ. This manuscript is reported in accordance with the Strengthening the Reporting of Observational Studies in Epidemiology (STROBE) [26] cross-sectional design reporting guideline and the (CHERRIES) checklist [27].

### 2.2 Study design

We conducted an anonymous, online, cross-sectional survey of all selected authors who have published in surgery journals indexed in Ovid MEDLINE over the past three years.

### 2.3 Sampling framework

A sample of corresponding authors who have published articles in various surgery journals between November 11, 2020 and October 15, 2023 were selected from a sample of general surgery journals as found on the National Library of Medicine (NLM) broad subject terms for indexed journals page (https://journal-reports.nlm.nih.gov/broad-subjects/). The NLM identifications (IDs) of the chosen journals were extracted. Subsequently, a search strategy was developed using these NLM IDs and used on Ovid MEDLINE. The resulting list of PubMed IDs (PMIDs) from this search was exported as a .csv file. An R script, constructed using the easyPubMed package [28], was executed to retrieve details such as authors' names, affiliated institutions, and email addresses. All authors who have published manuscripts of any type were incorporated into this study. No power analysis has been included as this study is based on a convenience sample and is primarily focused on descriptive work without inferential testing. The full search strategy can be accessed at the following link: https://osf.io/ta3pg.

### 2.4 Participant recruitment

The corresponding authors of articles published in the selected surgery journals were contacted to participate in our survey. Only surgery researchers and clinicians were eligible to complete the closed survey. SurveyMonkey was used to dispatch emails to the authors included in our sample. These potential participants received an email generated from an authorized recruitment script, which included a comprehensive explanation of the study's objectives, along with the survey link. Upon clicking the link, invitees were directed to read an informed consent form. Survey participants were asked to provide informed consent by clicking “Yes” to a question about consent agreement. After consenting, participants were then taken to the first page containing survey questions.

Duplicate email addresses within our list of authors, corresponding with those who have published multiple manuscripts in our sample, were deduplicated prior to sending our recruitment emails. Reminder emails were sent to individuals at intervals of one, two, and three weeks following the initial invitation email, and the survey was closed four weeks after the final reminder email was sent out. No financial compensation was provided for participation in this study, and respondents could skip any questions that they did not wish to answer.

### 2.5 Survey design

A survey was constructed and shared via the SurveyMonkey platform. Participants were presented with an initial screening question, followed by a series of multiple-choice questions surrounding their demographic details. The rest of the survey gathered information about each participants’ perceptions of CAIM and its perceived benefits and challenges through a series of multiple-choice, multi-select, and Likert scale questions. At the end of the survey, participants had the opportunity to answer an open-ended question about any additional comments they wished to share. A copy of the complete survey can be accessed at the following link: https://osf.io/svhzy.

### 2.6 Data management and analysis

This study did not have formal hypotheses. Fundamental descriptive statistics, such as counts and percentages, were generated by analyzing the quantitative data using GraphPad Prism 9. A thematic content analysis was also performed on the qualitative data. Twoauthors (JYN, BL) individually coded the received responses. The final codes were systematically organized and presented in distinct tables for reporting purposes.

## 3 Results

### 3.1 Search results

Of the 40 074 emails that were sent, 15 981 were unopened and 4102 bounced back. The bounced emails were excluded from response rate calculations. There were 599 total survey responses (corresponding to a 1.5 % response rate for opened and unopened emails and 3.1 % response rate for opened emails). On average, the survey took 8 minutes and 44 seconds to complete and had a 67 % completion rate. The raw, deidentified survey data can be accessed through this link: https://osf.io/6bma5.

### 3.2 Demographics

Of the survey respondents, most identified themselves as both clinicians and researchers (n = 331, 55.3 %), with some as solely researchers (n = 131, 21.9 %) and some as solely clinicians (n = 51, 8.5 %). Regarding geographical location according to the World Health Organization World Regions, most respondents were from Europe (n = 212, 41.9 %) and the Americas (n = 133, 26.3 %). Approximately half of respondents identified themselves as clinicians (n = 255, 50.3 %), followed by faculty members (n = 243, 47.9 %). Most respondents described themselves as senior researchers or clinicians (n = 279, 55.0 %), with over 10 years of starting their careers post formal education. Lastly, most respondents classified their primary research area to be clinical research (n = 353, 69.6 %). The full table of demographics information is found in Table 1.

**Table 1:** Demographics of survey respondents. CAIM: Complementary, alternative, and integrative medicine.

Sex (n = 506)	
Female	139 (27.5 %)
Male	361 (71.3 %)
Intersex	1 (0.2 %)
Prefer not to say	4 (0.8 %)
Prefer to self-describe	1 (0.2 %)
**Age (n = 507)**	
Under 18	0 (0 %)
18–24	4 (0.8 %)
25–34	92 (18.2 %)
35–44	179 (35.3 %)
45–54	113 (22.3 %)
55–64	73 (14.4 %)
65 or older	41 (8.1 %)
Prefer not to say	5 (1 %)
**Visible minority (n = 505)**	
Yes	94 (18.6 %)
No	388 (76.8 %)
Prefer not to say	23 (4.6 %)
**World Health Organization World Region (n = 506)**	
Africa	26 (5.1 %)
Americas	133 (26.3 %)
Eastern Mediterranean	21 (4.2 %)
Europe	212 (41.9 %)
South-East Asia	84 (16.6 %)
Western Pacific	18 (3.6 %)
Prefer not to say	12 (2.4 %)
**Profession (n = 507)**	
Clinician Student (e.g., medical student, nursing student, etc.)	9 (1.8 %)
Clinician (e.g., physician, nurse, etc.)	255 (50.3 %)
Graduate student	25 (4.9 %)
Postdoctoral fellow	36 (7.1 %)
Faculty member/Principal Investigator	243 (47.9 %)
Research support staff (E.g., research manager, research associate, technician)	21 (4.1 %)
Scientist in academia	94 (18.5 %)
Scientist in industry	3 (0.6 %)
Scientist in third sector (E.g., NGO, non-profit)	6 (1.2 %)
Government scientist	13 (2.6 %)
Other (please specify)	23 (4.5 %)
**Career stage (n = 506)**	
Graduate or clinician student	26 (5.1 %)
Early career researcher or clinician (<5 years of formally starting your career post formal education)	76 (15 %)
Mid-career researcher or clinician (5–10 years of starting your career post formal education)	125 (24.7 %)
Senior researcher or clinician (>10 years of starting your career post formal education)	279 (55.1 %)
**Primary research area (n = 410)**	
Clinical research	353 (86.1 %)
Preclinical research – in vivo	59 (14.4 %)
Preclinical research – in vitro	47 (11.5 %)
Health systems research	53 (12.9 %)
Health services research	70 (17.1 %)
Methods research	49 (12 %)
Epidemiological research	59 (14.4 %)
Other (please specify)	21 (5.1 %)
**Area of CAIM research (n = 407)**	
Mind-body therapies (e.g., meditation, biofeedback, hypnosis, yoga, tai chi, imagery, creative outlets)	42 (10.3 %)
Biologically based practices (e.g., vitamins and dietary supplements, botanicals, special foods and diets)	76 (18.7 %)
Manipulative and body-based practices (massage, chiropractic therapy, reflexology)	29 (7.1 %)
Biofield therapies (e.g., reiki, therapeutic touch)	5 (1.2 %)
Whole medical systems (e.g., Ayurvedic medicine, traditional Chinese medicine, acupuncture, homeopathy, naturopathic medicine)	29 (7.1 %)
I have never conducted any CAIM research	282 (69.3 %)
Other, please specify (e.g., methodology, CAIM professions, etc.)	9 (2.2 %)

### 3.3 Complementary, alternative, and integrative medicine

**Fig. 1:**
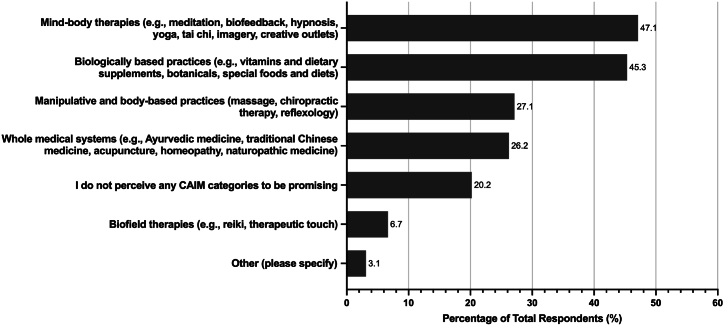
Areas of CAIM seen by respondents as the most promising for the speciality of surgery.

Most respondents had never conducted any CAIM research (n = 282, 69.3 %). Mind-body therapies (n = 212, 47.1 %) and biologically based practices (n = 204, 45.3 %) were seen as the most promising areas of CAIM in surgery (Fig. 1). Many clinicians declared that they have had patients who had sought counselling or disclosed using several different CAIMs, with the most common being biologically based practices (n = 230, 67.5 %), followed by similar rates for whole medical systems (n = 155, 45.5 %), mind-body therapies (n = 153, 44.9 %), and manipulative and body-based practices (n = 148, 43.4 %). Most clinicians responded that only 0–10 % of patients (n = 159, 46.8 %) disclosed or sought counselling on CAIM use in the past year. Many clinicians also responded that they had never practiced nor recommended CAIM to their patients (n = 112, 32.84 %). Most clinicians responded that they had never received formal (n = 244, 72.0 %) or supplemental (n = 199, 58.7 %) training in any areas of CAIM. Of the CAIM areas where clinicians had received training, the most common area was biologically based practices (n = 50, 14.8 % for formal training, and n = 74, 21.8 % for supplemental training). About half of clinicians responded that they have been asked about CAIM occasionally (n = 235, 52.2 %) outside of a clinical setting. The overwhelming majority of survey respondents declared that they would use academic literature to seek additional information on CAIM (n = 372, 82.7 %).

**Fig. 2:**
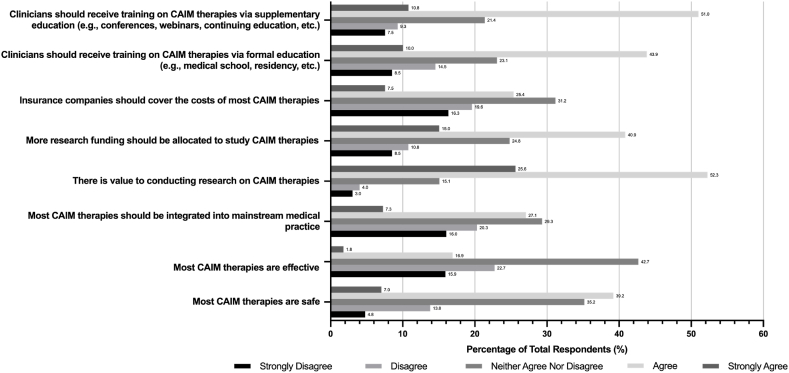
Degree of Agreement with General CAIM Statements.

The following responses about CAIM therapies were answered through a 5-point Likert scale, with the following options: “Strongly Disagree”, “Disagree”, “Neither Agree nor Disagree”, “Agree”, and “Strongly Agree”. When asked about the degree to which respondents would agree with general CAIM statements, many agreed (n = 156, 39.2 %) or strongly agreed (n = 28, 7.0 %) that most CAIM therapies are safe, however, they were less confident that CAIM therapies are effective (n = 153, 38.6 % disagree and n = 169, 42.7 % neither agree nor disagree) (Fig. 2). Many respondents also either agreed or strongly agreed that there is value in conducting CAIM research (n = 310, 77.9 %), that more funding should be allocated to CAIM research (n = 224, 55.9 %), and that clinicians should receive training on CAIM therapies through formal (n = 215, 52.9 %) and/or supplementary (n = 246, 61.8 %) education.

### 3.4 CAIM areas

Participants were then surveyed about the degree to which they agree with statements about mind-body therapies, biologically based therapies, manipulative and body therapies, biofield therapies, and whole medical systems (Table 2). Overall, the CAIM area that was perceived to be the most safe and effective was mind-body therapies, with 67 % agreeing or strongly agreeing that they are safe and 35 % agreeing that they are effective. In general, biofield therapies were perceived the least positively in response. Almost 30 % of survey respondents disagreed or strongly disagreed that biofield therapies are effective. Similarly, while many respondents had diverse ranges of agreement to whether they believe different CAIM areas should be integrated into mainstream medicine, there was the least support for biofield therapies. Participants also saw the least value in conducting research, increasing research funding, and receiving both formal and supplementary education for biofield therapies compared to the other four CAIM areas.

**Table 2:** Degree to which respondents agreed with statements regarding mind-body therapies, biologically based practices, manipulative and body therapies, biofield therapies, and whole medical systems.

Survey Question	CAIM Area	Strongly Disagree (%)	Disagree (%)	Neither Agree Nor Disagree (%)	Agree (%)	Strongly Agree (%)
Most [CAIM area] are safe.	Mind-body therapies	2.76	5.51	24.56	49.37	17.79
	Biologically based practices	6.80	17.13	35.52	35.77	4.79
	Manipulative and body therapies	7.61	15.23	35.28	37.06	4.82
	Biofield therapies	7.09	9.62	48.10	28.61	6.58
	Whole medical systems	9.39	12.94	40.86	31.73	5.08
Most [CAIM area] are effective.	Mind-body therapies	6.30	12.85	45.34	28.97	6.55
	Biologically based practices	9.60	23.23	40.40	22.98	3.79
	Manipulative and body therapies	8.86	13.16	44.56	29.62	3.80
	Biofield therapies	16.71	19.24	49.11	12.41	2.53
	Whole medical systems	13.62	15.94	44.99	21.59	3.86
Most [CAIM area] should be integrated into mainstream medical practice.	Mind-body therapies	8.54	17.34	36.43	30.40	7.29
	Biologically based practices	12.09	18.64	33.25	30.73	5.29
	Manipulative and body therapies	10.15	20.05	36.04	28.43	5.33
	Biofield therapies	18.02	22.59	43.65	12.94	2.79
	Whole medical systems	16.50	18.27	34.01	26.14	5.08
There is value to conducting research on [CAIM area].	Mind-body therapies	3.27	6.03	19.85	52.76	18.09
	Biologically based practices	4.29	4.04	18.69	56.57	16.41
	Manipulative and body therapies	6.60	6.35	22.59	53.05	11.42
	Biofield therapies	11.39	12.66	36.71	33.16	6.08
	Whole medical systems	9.44	5.61	21.68	49.49	13.78
More research funding should be allocated to study [CAIM area].	Mind-body therapies	8.04	10.55	27.64	39.70	14.07
	Biologically based practices	6.85	7.61	29.44	43.65	12.44
	Manipulative and body therapies	9.92	11.20	31.04	38.42	9.41
	Biofield therapies	15.70	15.44	42.53	18.99	7.34
	Whole medical systems	12.69	10.15	31.47	32.99	12.69
Insurance companies should cover the costs of most [CAIM area].	Mind-body therapies	12.59	15.62	38.29	25.69	7.81
	Biologically based practices	13.96	19.29	38.07	22.84	5.84
	Manipulative and body therapies	13.96	15.48	39.34	26.40	4.82
	Biofield therapies	20.51	21.27	42.78	11.90	3.54
	Whole medical systems	17.51	17.51	34.77	24.62	5.58
Clinicians should receive training on [CAIM area] via formal education (e.g., medical school, residency, etc.)	Mind-body therapies	7.81	16.37	26.20	41.31	8.31
	Biologically based practices	8.33	13.89	23.74	47.22	6.82
	Manipulative and body therapies	10.63	15.44	31.39	36.46	6.08
	Biofield therapies	15.44	20.76	40.25	20.25	3.29
	Whole medical systems	14.97	14.21	27.41	37.06	6.35
Clinicians should receive training on [CAIM area] via supplementary education (e.g., conferences, webinars, continuing education, etc.)	Mind-body therapies	7.54	9.55	25.13	48.99	8.79
	Biologically based practices	7.83	9.09	26.01	49.24	7.83
	Manipulative and body therapies	9.11	10.89	35.44	38.73	5.82
	Biofield therapies	13.92	16.71	41.27	24.30	3.80
	Whole medical systems	12.69	11.68	29.19	39.09	7.36

### 3.5 Comfort with counselling about or recommending CAIM

**Fig. 3:**
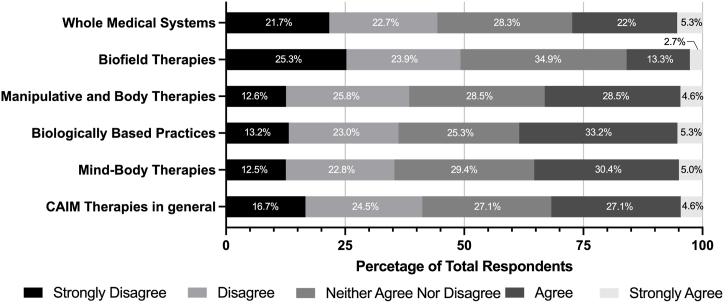
Degree of comfort with counselling patients about CAIM therapies.

**Fig. 4:**
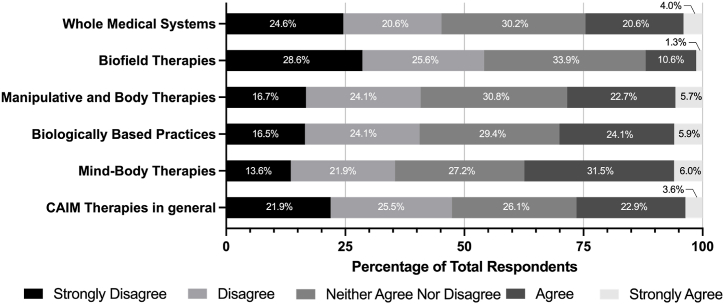
Degree of comfort with recommending CAIM therapies to patients.

Overall, respondents were generally not comfortable with counselling about different CAIM therapies with their patients (Fig. 3). More clinicians were not comfortable with counselling (n = 126, 41.2 %) about CAIM therapies in general compared to clinicians who were (n = 97, 31.7 %). Survey respondents generally expressed discomfort with recommending CAIM therapies, with respondents being the most comfortable with recommending mind-body therapies (n = 107, 37.5 %) (Fig. 4). There was discomfort with recommending CAIM therapies in general (n = 145, 47.4 %) and clinicians were the least comfortable with recommending biofield therapies (n = 163, 54.2 %).

### 3.6 Benefits and challenges

**Fig. 5:**
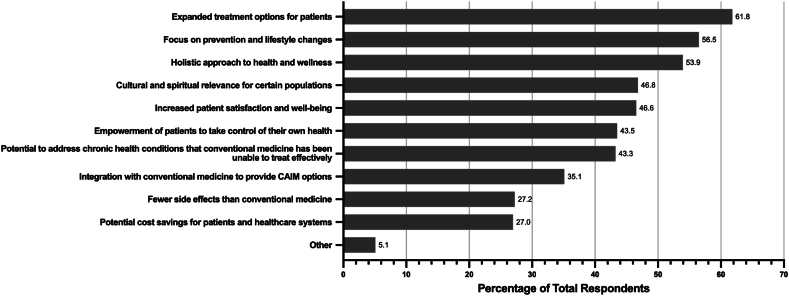
Perceived benefits associated with CAIM within the speciality of surgery.

**Fig. 6:**
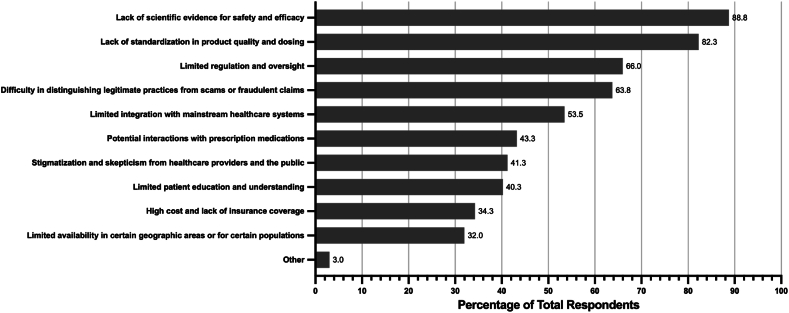
Perceived challenges associated with CAIM within the speciality of surgery.

The greatest benefits that respondents perceived to be associated with CAIM were expanded treatment options for patients (n = 243, 61.8 %), a focus on prevention and lifestyle changes (n = 222, 56.5 %), and a holistic approach to health and wellness (n = 212, 52.9 %) (Fig. 5). Conversely, the greatest challenges that respondents perceived to be associated with CAIM were a lack of scientific evidence for safety and efficacy (n = 355, 88.8 %), a lack of standardization in product quality and dosing (n = 329, 82.3 %), limited regulation and oversight (n = 264, 66.0 %), and difficulty in distinguishing legitimate practices from scams or fraudulent claims (n = 255, 63.8 %) (Fig. 6).

### 3.7 Thematic analysis

In total, 22 codes were devised from the 63 open-ended responses. From these codes, 4 major themes were identified. The first was “benefits of CAIM”, which included four subthemes involving support of CAIM in general, support of specific CAIMs, and benefits for patients. The next theme was “integrative use of CAIM”, including two subthemes regarding how CAIM can be used alongside conventional treatments. Another theme was “concerns regarding the negative impacts of CAIM”, which included seven subthemes regarding the potentially predatory or fraudulent nature of some therapies, harm associated with insufficient training, a lack of standardization, and general opposition of its usage. The last theme was “insufficiency of CAIM research and education”, which included seven subthemes regarding a need for a greater evidence basis and patient or clinician education. The theme with the most coded responses was regarding the insufficiency of CAIM research and education. A copy of the full coding and thematic analysis data can be accessed through the following link: https://osf.io/pq3vt.

## 4 Discussion

The objective of this study was to investigate the existing perspectives of both surgical researchers and clinicians regarding CAIM. To the best of our knowledge, there have been no international studies that have collected the perceptions of surgery clinicians and researchers on CAIM. Our findings are consistent with current studies within the literature pertaining to other medical specialties. Studies have been conducted in the fields of psychiatry, oncology, and neurology that surveyed researchers and clinicians about their perceptions of CAIM. All three fields found very similar results regarding statements about different CAIM areas, with the most positive perceptions on CAIM research and education. Similarly to this study, the three fields also found mind-body therapies to be the most positively regarded [[29], [30], [31]]. However, while biologically based practices were found to be the second most positively regarded for this study as well as the oncology and psychiatry studies, the neurology study had more negative perceptions of this CAIM area [[29], [30], [31]]. In a study performed on the perceptions of CAIM among Swedish surgery healthcare professionals, many interviewees mentioned concerns surrounding the evidence-basis of CAIM and the lack of knowledge surrounding the field [24]. Another study analysed the attitudes of CAIM among surgeons in Hungary, which yielded similar results regarding increased CAIM training through formal education [19]. As well, our results paralleled another study which found that surgery healthcare practitioners had more negative perceptions about biofield therapies and whole medical systems [25]. Compared to other types of CAIM treatments, the most research has been conducted on the attitudes of surgery clinicians on the use of acupuncture and acupressure in perioperative care. Similar to our study, these studies found that surgery clinicians had positive attitudes towards acupuncture and acupressure and supported its evidence-based usage, despite having little education on these therapies [32].

It was found that some areas of CAIM are more positively regarded than others, with the most positive perceptions of mind-body therapies and most negative perceptions of biofield therapies. Overall, there were greater positive perceptions of CAIM areas that included therapies that could be used complementary to conventional treatments (e.g., mind-body therapies) compared to those that are more frequently used alternatively to conventional treatments (e.g., whole medical systems). Mind-body therapies encompass treatments such as yoga, hypnosis, and meditation. This CAIM area may have more positive perceptions due a greater evidence basis and a lower risk of adverse interactions with conventional therapies. Several studies on the use of various mind-body therapies pre-operatively found that patients had lower levels of post-operative pain [33]. In contrast, biofield therapies may have more negative perceptions due to its comparatively lower evidence basis [34]. This lack of evidence-based research could also contribute to more negative perceptions that surgery researchers and clinicians have toward integrating CAIM therapies into mainstream medical practice. Additionally, although many survey respondents support CAIM research and increasing the funding of it, the open-ended responses also show that respondents believe CAIM research should only be funded or implemented if there is a realistic expectation for it to be beneficial. More specifically, studies on CAIM therapies should undergo the same methodological rigor as other areas of medicine to ensure their benefit for patients. Previous studies have shown that there is a high degree of heterogeneity in the quality of CAIM research, with some studies being more methodologically rigorous than others [23].

Though nearly half of all survey respondents agreed that most CAIM therapies are safe, less than a fifth agreed that they are effective. This may be due to a lack of evidence-based research confirming the benefits of CAIM. Some of the most common CAIM treatments used in surgery and perioperative care are massage therapy, herbal medicines, acupuncture, prayer, yoga, and relaxation therapy [35]. Within these treatments, there is substantially more evidence from methodologically sound studies for massage therapy, acupuncture, and yoga [21,[36], [37], [38]]. The thematic analysis also showed concerns surrounding the use of CAIM with conventional treatments. This presents a need for clinicians to be better educated on CAIM therapies as some therapies may have adverse effects with conventional medications that clinicians should be informed about prior to beginning surgery. Moreover, without CAIM training or education, some surgery clinicians may be dismissive of CAIM therapies despite there being evidence-based research supporting their safety and efficacy.

Half of clinicians reported that only 0–10 % of patients disclosed the use of CAIM therapies within the past year. A previous study has shown that while the prevalence of CAIM use in cancer patients ranged from 11 % to 95 %, only 20 %–77 % of these patients disclosed their use to their care providers [39]. There are diverse reasons for non-disclosure of CAIM therapies, which include lack of inquiry from medical providers, fear of provider disapproval, perception of disclosure as unimportant, and belief that providers lacked CAIM knowledge [40,41]. Though herbal medications remain a popular option among patients undergoing surgery [9], many studies have shown that non-disclosure of ingestible and natural CAIM therapies is more prevalent than non-disclosure of physical therapies [40,42]. This may be because the use of biologically based therapies (e.g., herbal supplements) is more discouraged as there is a greater risk for adverse interaction with conventional medical treatments [18].

While the majority of survey respondents have never had formal or supplemental training on CAIM, many agreed that clinicians should receive formal or supplemental training on most CAIM areas. This is consistent with researcher and physician attitudes from other studies on CAIM, where many clinicians or researchers believe that CAIM training and education is inadequate [43]. To better support patients’ interests in using CAIM therapies for reducing perioperative pain and anxiety, it is important for surgery clinicians to be educated on its usage and effects. One demographic that has been frequently targeted for CAIM education is medical students. Several studies have shown positive results when integrating CAIM into medical school curricula [44,45]. Moreover, strategies have also been developed to improve the integration of CAIM instruction into professional schools. For example, the Mayo Clinic has created an integrative medicine education program for its medical students and physicians, using a combination of didactic and hands-on approaches [46]. CAIM education has also been added as a competency to the medical school curriculum in Switzerland [47]. Other strategies include greater top-down support for CAIM from institutional leaders, building relationships with reputable CAIM practitioners to develop instructional materials, as well as sustaining CAIM initiatives independent of financial support [48].

### 4.1 Strengths and limitations

Our study has several strengths. The cross-sectional survey design is time efficient, as the survey was administered on the SurveyMonkey platform and all data were collected at a single time point. Additionally, the international scope and selection of participants enhances the generalizability of our findings regarding surgery researcher perceptions of CAIM. Furthermore, participant contact information was obtained through NLM categorization, streamlining the process. As well, emails were only sent to researchers who had published within the last three years to minimize the chances of using invalid or inactive email addresses. Lastly, sending multiple reminders at one-week intervals with a four-week cool down period improved participant response rates, thereby increasing the sample size.

Regarding limitations, as the survey was administered in the English language, our findings may not be entirely representative of researchers and clinicians who are not English speakers. Moreover, because we used a sampling method that retrieves contact information from academic journals, we expected a higher response rate from researchers compared to clinicians. As well, due to our sampling method, our sample of clinicians is also not generalizable to clinicians who do not conduct research. We also anticipated a low survey participation rate due to various factors. These include the possibility that some participants changed their affiliations, lost access to their email, and were on vacation, retired, or passed away. These factors could introduce nonresponse bias into our results. Moreover, our survey findings may be skewed toward individuals who are already familiar or have strongly positive or negative attitudes toward CAIM, as surgery researchers and clinicians with limited interest or knowledge of this topic may have been less inclined to participate. Lastly, participants could experience recall bias, where their responses may be affected by the differing accuracy and knowledge with which they remember their own experiences, given the survey's reliance on self-reporting.

## 5 Conclusions

This study investigated the knowledge and attitudes of surgery clinicians and researchers on CAIM. There were varying degrees of acceptance and attitudes across different CAIM areas, with mind-body therapies perceived to be the most promising and biofield therapies perceived to be the least. Our study provides valuable insight in understanding surgery clinician and researcher knowledge and perception of CAIM and establishes a need to improve CAIM training and education for these individuals. The information gathered from this study can be used as a foundation for creating educational resources and training programs that better inform researchers and clinicians about CAIM and address their knowledge gaps and barriers.

## CRediT authorship contribution statement

**Jeremy Y. Ng:** Writing – review & editing, Writing – original draft, Supervision, Project administration, Methodology, Investigation, Formal analysis, Data curation, Conceptualization. **Brenda X. Lin:** Writing – review & editing, Writing – original draft, Investigation, Formal analysis, Data curation. **Holger Cramer:** Writing – review & editing, Supervision, Methodology, Investigation, Formal analysis, Data curation, Conceptualization.

## Ethics approval and consent to participate

This study received approval from the University Tübingen Research Ethics board (REB Number: 389/2023BO2).

## Consent for publication

All included study participants consented to participating in this study and to have their survey responses published in a peer reviewed journal.

## Availability of data and materials

All data and materials associated with this study have been posted on the Open Science Framework and can be found here: https://doi.org/10.17605/OSF.IO/8NUGQ.

## Funding

This study was unfunded. We acknowledge support from the Open Access Publication Fund of the University of Tübingen.

## Declaration of competing interest

The authors declare that they have no known competing financial interests or personal relationships that could have appeared to influence the work reported in this paper.

## List of Abbreviations

CAIM: complementary, alternative, and integrative medicine
CHERRIES: Checklist for Reporting Results of Internet E-Surveys
DOI: digital object identifier
MEDLINE: Medical Literature Analysis and Retrieval System Online
NLM: National Library of Medicine
OSF: Open Science Framework
PMID: PubMed identifier
REB: Research Ethics Board
STROBE: Strengthening the Reporting of Observational Studies in Epidemiology
